# FtBPM3 modulates the orchestration of FtMYB11‐mediated flavonoids biosynthesis in Tartary buckwheat

**DOI:** 10.1111/pbi.13587

**Published:** 2021-05-02

**Authors:** Mengqi Ding, Kaixuan Zhang, Yuqi He, Qian Zuo, Hui Zhao, Ming He, Milen I. Georgiev, Sang Un Park, Meiliang Zhou

**Affiliations:** ^1^ Institute of Crop Sciences Chinese Academy of Agricultural Sciences Beijing China; ^2^ Department of Crop Science College of Agriculture & Life Sciences Chungnam National University Daejeon Korea; ^3^ School of Life Sciences Hunan University of Science and Technology Xiangtan Hunan China; ^4^ Laboratory of Metabolomics The Stephan Angeloff Institute of Microbiology Bulgarian Academy of Sciences Plovdiv Bulgaria; ^5^ Center of Plant Systems Biology and Biotechnology Plovdiv Bulgaria; ^6^ Department of Smart Agriculture Systems Chungnam National University Daejeon Korea

**Keywords:** BTB, POZ AND MATH DOMAIN proteins, Jasmonate signalling, MYB transcriptional repressor, Secondary metabolite, 26S proteasome

Tartary buckwheat (*Fagopyrum tataricum*, TB) is rich in bioactive flavonoids, which have a variety of biological activities (Ghorbani, 2017). Jasmonates (JAs) are essential phytohormones, which play key roles in regulating the formation of numerous secondary metabolites, including flavonoids rutin (Li *et al.,* 2019; Zhou and Memelink, 2016). It has been reported that JAs could induce flavonoids accumulation and identified a class of JAs‐responsive R2R3‐MYB TFs, such as FtMYB11, a repressor of rutin biosynthesis in TB (Zhang *et al.,* 2018; Zhou *et al.,* 2017). JAs lead to FtMYBs degradation by 26S proteasome pathway (Zhang *et al.,* 2018). However, the posttranslational regulation of these FtMYBs has not been reported.

Recently, we reported genome re‐sequencing data of 510 TB accessions and genome‐wide association study (GWAS) of three main flavanols, among which the kaempferol‐3‐*O*‐rutinoside (KC) is stable and less affected by environmental factors compared with quercetin and rutin (Zhang *et al.,* 2021). Four associated loci passing the threshold *P* < 1 × 10^−5^ were identified, and three associated loci repetitively in two years (Figure 1a). However, no known flavonoids metabolism genes were identified and no genes were significantly induced by JA (data not shown). Interestingly, *FtBPM3* (*FtPinG0808645400*), a homolog of *Arabidopsis* BTB‐POZ/MATH (BPM) E3 ligase, which functions as an important regulator of JA‐responsive TFs activity and stability (Chico *et al.,* 2020; Li *et al.,* 2021) was associated with Ft8:31558491. Further analysis showed KC content (Figure 1b) and *FtBPM3* expression (Figure 1c) was higher in the A/A genotype than the A/C genotype, suggesting *FtBPM3* was a candidate gene controlling flavonoids biosynthesis in TB.

**Figure 1:**
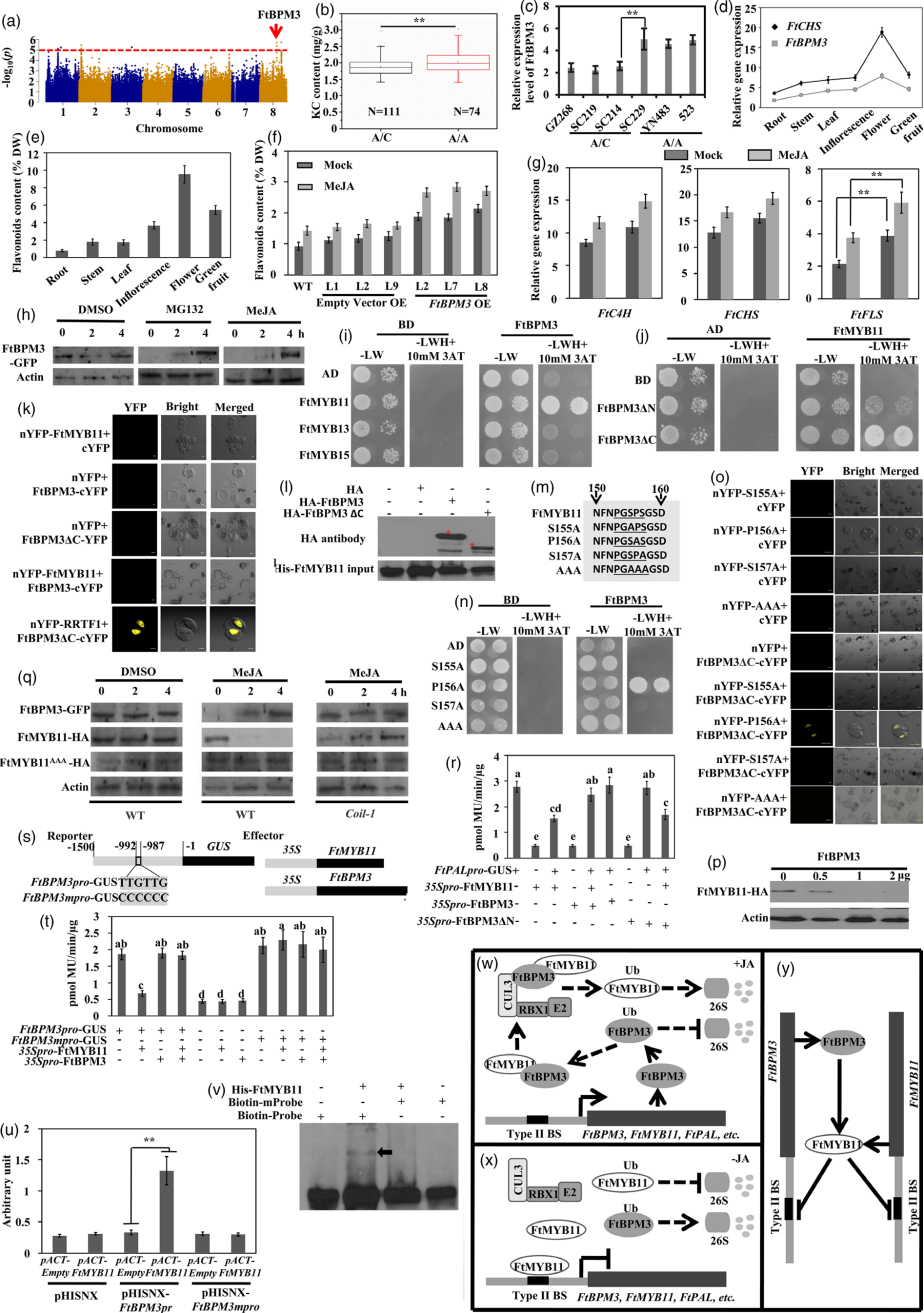
FtBPM3 modulates the orchestration of FtMYB11‐mediated flavonoids biosynthesis in Tartary buckwheat. (a) Local Manhattan plot of association loci with the KC contents. (b) Box plots show KC contents in two haplotypes. (c) The expression levels of FtBPM3 in A/A and A/C type. (d‐e) *FtCHS* and *FtBPM3* expression patterns (d) and flavonoids concentration (e) in different tissues. DW, Dry weight. (f‐g) Flavonoids content (f) and the expression levels of *FtC4H*, *FtCHS* and *FtFLS* (g) in different types of TB hairy roots in response to 50 μΜ MeJA for 4 h. (h) Immunoblot analysis of FtBPM3‐HA protein levels in *FtBPM3OE* lines. (i‐l) Y2H (i‐j), BIFC (k) and Pull‐down assay (l) of FtMYB11 and FtBPM3 or its derivatives. (m) FtMYB11 SBC‐like motif and derivatives. (n‐o) Y2H and BIFC assay of FtBPM3 and FtMYB11^P156A^. (p) Immunoblot analysis of FtMYB11‐HA protein levels in Arabidopsis suspension protoplasts expressing *35S::FtBPM3*. (q) Immunoblot analysis of FtBPM3‐GFP, FtMYB11‐HA and FtMYB11^AAA^‐HA protein levels in WT or *Coil‐1* Arabidopsis suspension protoplasts treated with 50 μm MeJA. (r) Trans‐activation assays of *FtPAL* promoter by FtMYB11, FtBPM3 or FtBPM3ΔN. (s‐t) Trans‐activation assays of *FtBPM3pro‐GUS* or *FtBPM3mpro‐GUS* by FtMYB11 and/or FtBPM3. (u) Transcriptional activity assays of FtMYB11 binding to the *FtBPM3pro* or *FtBPM3mpro* fragments in yeast. (v) EMSA of a wild type or mutated probe of *FtBPM3pro* with FtMYB11. (w‐y) Model of the fine‐tuned ‘ping‐pong’ regulatory mechanism between FtMYB11 and FtBPM3 activity in JAs signalling pathway. JA‐pathway activation stabilizes FtBPM3 (w). Without JA, the ubiquitination of FtBPM3 is degraded by the 26S proteasome (x), FtBPM3 targets FtMYB11 for degradation (y). Data in (b), (c), (g) and (u) are means ± SE of three biological repeats (Student’s *t*‐test, *P < 0.05, **P < 0.01).

To test whether the expression pattern of *FtBPM3* is consistent with TB flavonoids biosynthesis, the expression patterns of *FtBPM3* and *CHS* (chalcone synthase), a key gene in TB flavonoids biosynthesis and the flavonoids content in different tissues TB plant were studied. The expression of *FtBPM3* appeared closely associated with *CHS* (Figure 1d) and flavonoids content (Figure 1e), indicating the expression of *FtBPM3* is probably involved in flavonoids accumulation. The flavonoids levels (Figure 1f) and the expression of three genes (*FtC4H*, *FtCHS* and *FtFLS*) (Figure 1g) in the *FtBPM3* overexpressing hairy roots lines were significantly higher than those in control. MeJA (methyl jasmonate) treatment drastically increased the accumulation of flavonoids in *FtBPM3* overexpressing transgenic lines (Figure 1f), indicating that FtBPM3 protein is stable in response to MeJA. Immunoblot analysis of FtBPM3‐HA protein levels in *FtBPM3* overexpressing lines revealed that both MeJA and MG132 drastically increased FtBPM3‐HA accumulation (Figure 1h), indicating FtBPM3 protein is subject to 26S proteasome‐mediated degradation. Taken together, these results demonstrated FtBPM3 protein accumulation promotes JA‐induced flavonoids biosynthesis.

Yeast two‐hybrid (Y2H) assays were used to examine whether FtBPM3 assembles with JA‐responsive subgroup 4 MYB repressors and found FtBPM3 interacts strongly with FtMYB11, while FtMYB13 and FtMYB15 did not (Figure 1i). And this interaction requires the N‐terminal MATH domain of FtBPM3 (Figure 1j). However, no YFP signal was observed by bimolecular fluorescence complementation (BiFC) assay in the combination of full‐length FtBPM3 and FtMYB11, but FtBPM3ΔC lacking a BTB‐POZ domain does (Figure 1k), indicating that FtBPM3 probably targets FtMYB11 for protein degradation. Pull down assay found direct physical interaction between HA‐FtBPM3 and His‐FtMYB11 (Figure 1l). These results consistently support the direct interaction between FtBPM3 and FtMYB11.

It has been reported that *Arabidopsis* BPM proteins interact with their target proteins through the speckle‐type POZ protein (SPOP)‐binding consensus (SBC)‐like motif ϕ‐π‐S‐X‐S/T (ϕ, nonpolar; π, polar; X, any amino acid) (Morimoto *et al.,* 2017). To test whether SBC‐like motif in FtMYB11 was responsible for the recognition of FtMYB11 by FtBPM3, the interaction between FtBPM3 and FtMYB11 derivatives (Figure 1m), was tested by Y2H assays. FtMYB11^S155A^, FtMYB11^S157A^ and FtMYB11^AAA^ lost interaction with FtBPM3, while FtMYB11^P156A^ did not affect the interaction (Figure 1n). BiFC assay showed a YFP signal only observed in the nucleus of *Arabidopsis* protoplasts upon co‐expression of FtBPM3ΔC‐cYFP with nYFP‐FtMYB11^P156A^ (Figure 1o). These results indicate that the SBC‐like motif of FtMYB11 is hence sufficient for the interaction with FtBPM3.

The interaction between FtBPM3 and FtMYB11 suggested that FtMYB11 could be targets of CUL3^FtBPM3^ E3 ubiquitin ligases. Thus, we sought to examine whether increment of FtBPM3 expression affects FtMYB11 stability using a transient expression assay in *Arabidopsis* protoplasts. Total proteins were extracted from protoplasts co‐transformed with FtMYB11‐HA, with or without FtBPM3‐His and subjected to immunoblot analysis. The addition of FtBPM3 led to FtMYB11 degradation, whereas the internal control was not significantly affected (Figure 1p). Since both FtBPM3 and FtMYB11 are responsive to JA at protein levels, we then tested whether their stability was associated with the 26S proteasome in a JA‐dependent manner in wild type (WT) and *Coil‐1* protoplasts. JA treatment significantly decreased FtMYB11‐HA protein level, but not for FtMYB11^AAA^‐HA, and drastically increased the accumulation of FtBPM3‐His with time in WT background (Figure 1q). However, the level of two proteins was not changed under JA treatment in *Coil‐1* background, indicating FtBPM3 targets FtMYB11 for degradation depending on JA signalling.

Our previous results showed that FtMYB11 directly represses the *FtPAL* gene expression (Zhou *et al.,* 2017). To further elucidate the effect of FtBPM3 on the activity of FtMYB11, *Arabidopsis* protoplast trans‐activation assays were performed. The co‐transformation of *FtPALpro‐GUS* reporter and *35S::FtMYB11* effector resulted in strong repression (Figure 1r). The addition of the *35S::FtBPM3* effector resulted in the repression of FtMYB11 activity, but *35S::FtBPM3ΔN* has no effect. These results support the above protein interaction and FtBPM3 could regulate FtMYB11 activity.

Sequence analysis showed the promoter of *FtBPM3* contains one type II element, which was recognized by FtMYB11 (Zhou *et al.,* 2017). *Arabidopsis* trans‐activation assays showed FtMYB11 repressed the *FtBPM3pro‐GUS* reporter and had no effect on *FtBPM3mpro‐GUS* (Figure 1s,t), indicating this type II element is important for transcriptional repression. The addition of FtBPM3 released the repression activity of FtMYB11 (Figure 1t), indicating FtMYB11 may directly repress *FtBPM3* gene expression *via* binding to the type II element. Yeast one‐hybrid (Y1H) assays identified the direct interactions between FtMYB11 and *FtBPM3* promoter fragment, but not with its derivative (Figure 1u). Electrophoretic mobility shift assay (EMSA) showed incubation of the wild type with His‐FtMYB11 produced shifts, while the mutant 80‐bp probes did not result in shifts (Figure 1v). These results illustrated that FtMYB11 could directly repress the *FtBPM3* gene expression.

In summary, we provide a new framework to understand the fine‐tuned ‘ping‐pong’ regulatory mechanism between FtMYB11 and FtBPM3 activity. We uncovered a negative feedback regulatory loop of FtMYB11 protein levels mediated by the E3 ligase CUL3^FtBPM3^ that facilitates termination of FtBPM3 mRNA accumulation to avoid the overaccumulation of FtBPM3 (Figure 1w‐y). This ‘ping‐pong’ mechanism is necessary for resetting JA signalling and to avoid harmful runaway responses, which optimize plant fitness and provide a theoretical basis for the cultivation of TB with high flavonoid content.

## Conflict of interest

The authors declare no conflicts of interest.

## Author contributions

M.Z., M.I.G. and S.U.P conceived and supervised the research. M.D., K.Z., Q.Z. and Y.H conducted the experiments. H.Z. and M.H analysed the data. M.D., M.I.G., S.U.P., M.Z. and Y.H wrote the paper.
