# Correction to “FtBPM3 modulates the orchestration of FtMYB11‐mediated flavonoids biosynthesis in Tartary buckwheat”

**DOI:** 10.1111/pbi.70698

**Published:** 2026-05-31

Ding, M., Zhang, K., He, Y., Zuo, Q., Zhao, H., He, M., Georgiev, M. I., Park, S. U. and Zhou, M. (2021) FtBPM3 modulates the orchestration of FtMYB11‐mediated flavonoids biosynthesis in Tartary buckwheat. *Plant Biotechnol. J*, **19**, 1285‐1287.

In the above article, the authors would like to correct Figure 1l and the labels of Figures 1h and 1k, as well as the description of Figure 1q. In Figure 1l, the input image of the pull‐down assay was mistakenly taken from Figure 1v; the correct Figure 1l is shown below. In Figure 1h, the first panel label should be ‘FtBPM3‐HA’ instead of ‘FtBPM3‐GFP’. In Figure 1k, the third and last panel labels should be ‘nYFP+FtBPM3ΔC‐cYFP’ and ‘nYFP‐FtMYB11+FtBPM3ΔC‐cYFP’, instead of ‘nYFP+FtBPM3ΔC‐YFP’ and ‘nYFP‐RRTF1+FtBPM3ΔC‐cYFP’, respectively. In the main text describing Figure 1q, ‘FtBPM3‐His’ should be ‘FtBPM3‐GFP’.

These errors do not affect the results.

**Figure:**
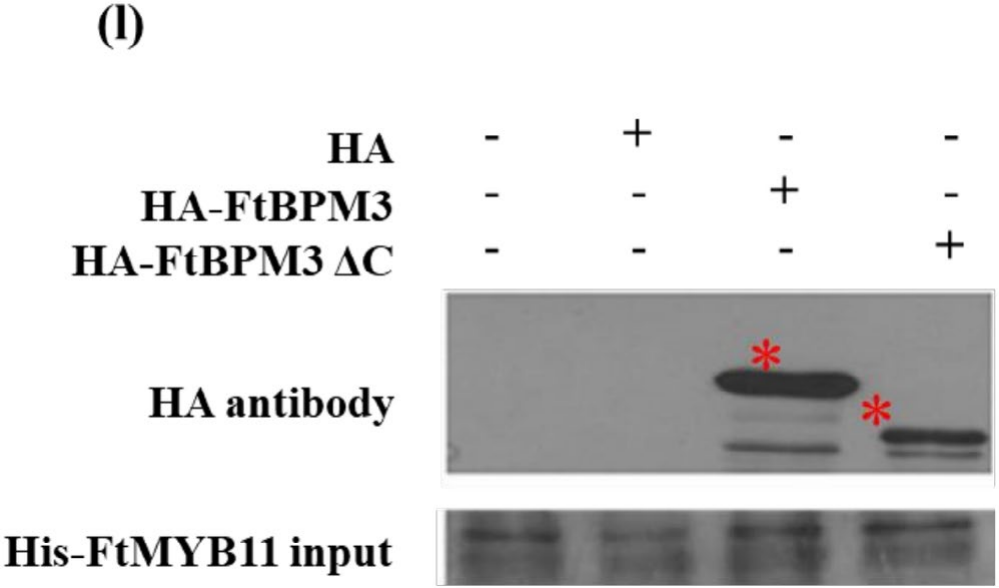
**FIGURE 1** (l) Pull‐down assay of FtMYB11 and FtBPM3 or its derivatives.

We apologize for this error.

